# Long-Term Monitoring of Fresco Paintings in the Cathedral of Valencia (Spain) Through Humidity and Temperature Sensors in Various Locations for Preventive Conservation

**DOI:** 10.3390/s110908685

**Published:** 2011-09-08

**Authors:** Manuel Zarzo, Angel Fernández-Navajas, Fernando-Juan García-Diego

**Affiliations:** 1 Department of Applied Statistics, Operations Research and Quality, Universidad Politécnica de Valencia, Camino de Vera s/n, 46022 Valencia, Spain; E-Mail: mazarcas@eio.upv.es; 2 Department of Applied Physics (U.D. Agriculture Engeering), Universidad Politécnica de Valencia, Camino de Vera s/n, 46022 Valencia, Spain; E-Mail: afnavajas@fis.upv.es

**Keywords:** microclimate, art conservation, diagnosis, sensor, multivariate monitoring

## Abstract

We describe the performance of a microclimate monitoring system that was implemented for the preventive conservation of the Renaissance frescoes in the apse vault of the Cathedral of Valencia, that were restored in 2006. This system comprises 29 relative humidity (*RH*) and temperature sensors: 10 of them inserted into the plaster layer supporting the fresco paintings, 10 sensors in the walls close to the frescoes and nine sensors measuring the indoor microclimate at different points of the vault. Principal component analysis was applied to *RH* data recorded in 2007. The analysis was repeated with data collected in 2008 and 2010. The resulting loading plots revealed that the similarities and dissimilarities among sensors were approximately maintained along the three years. A physical interpretation was provided for the first and second principal components. Interestingly, sensors recording the highest *RH* values correspond to zones where humidity problems are causing formation of efflorescence. Recorded data of *RH* and temperature are discussed according to Italian Standard UNI 10829 (1999).

## 1. Introduction

### 1.1. Thermo-Hygrometric Conditions in Museums and Churches

The indoor thermo-hygrometric conditions of a museum should be appropriate for the conservation and display of the collections inside, such that deterioration is either stopped or at least slowed to acceptable rates. Potential risks are related to imbalance in the temperature and humidity, generated by different factors such as heating, air conditioning, ventilating system, exchange of outside air, or large visitors numbers [1]. Attempting to assess *in situ* the complexity of these risks, different studies have monitored microclimate parameters (e.g., air temperature, relative humidity, specific humidity, dew point, *etc.*) inside museums such as the British Museum in London [2] or the Chiericati Palace Municipal Museum in Vicenza, Italy [3]. A similar study developed along several years was conducted in the Uffizi Gallery in Florence, Italy. Marked thermo-hygrometric gradients were found, which might become harmful to the exhibitions in the long term [4].

Churches and cathedrals contain valuable works of art, and the microclimate requirements are similar as those in museums. Different researchers have monitored the temperature (T^a^) and relative humidity (*RH*) in churches located in Russia [5], Italy [6,7] and Cyprus [8]. The latter work identified large diurnal and seasonal variations.

Apart from microclimate parameters, other reported studies have thoroughly characterized the air quality inside four European museums by measuring concentration of damaging pollutant gases, deposition and origin of the suspended particulate matter, as well as the concentration of airborne microorganisms [9–13].

### 1.2. Preventive Conservation of Fresco Paintings by Microclimate Monitoring

Fresco is a method of painting on freshly plastered walls or ceilings with powdered pigments mixed with water. The late medieval period and the Renaissance saw the most prominent use of this technique, particularly in Italy, where most churches and many government buildings still feature fresco decoration.

Temperature and humidity changes can affect the conservation of frescoes. Ideally, the wall temperature should be at any point the same as the air temperature on the wall surface and in the immediate proximity. If it is different, it generates an air flow along the wall surface that increases the aerodynamic deposition of airborne particles and wall soiling. The natural ventilation and turbulence indoors also affect the transport and diffusion of airborne pollutants. Different works have characterized the distribution of thermal and hygrometric parameters, as well as the turbulence indoors, in order to study the interactions between the indoor atmosphere and walls supporting frescoes or mural paintings [14–16]. The impact of other adverse factors such as heating, lighting, solar radiation, or people were also discussed.

Vertical gradients of temperature (*i.e.*, variations with height) generate air flows along the surface of frescoes that increase the aerodynamic deposition of airborne particles and soiling. Thus, weak gradients of temperature and *RH* are desirable for conservation purposes given that these conditions lead to little deposition of pollutants on the frescoes.

Leonardo’s “Last Supper” is a famous mural painting located in the monastery of Santa Maria delle Grazie in Milan. Some authors [17] analyzed the thermo-hygrometric gradients which exist between the mural and surrounding air as well as the deposition processes that are induced. The internal stresses forced on the painting by the lighting system, the central heating, and the presence of visitors were also discussed. In a later work, these authors measured the main environmental parameters and studied the microclimate dynamics inside the Sistine Chapel of the Vatican in order to assess the factors (e.g., deposition of pollutants, mechanical stresses, microfractures, condensation and evaporation cycles in the micropores, *etc.*) which may cause dangerous microphysical processes affecting the famous Michelangelo’s frescoes [18].

### 1.3. Renaissance Frescoes at the Cathedral of Valencia, Spain

The construction of the metropolitan basilica cathedral of St. Mary in Valencia began in 1262. Two hundred years later, the whole decoration of the apse was burnt by an accidental fire that destroyed the vault paintings. Several restoration attempts failed. In 1472, the Valencian bishop Rodrigo Borgia (later Pope Alexander VI) traveled from his residence in Rome to Valencia with the idea of bringing to the cathedral the new art movement coming up in Italy, the Renaissance. For this purpose, he brought two Italian painters, Francesco Pagano and Paolo da San Leocadio, who painted in fresco each of the severies between the ribs of the vaulted roofs above the presbytery. In 1674 the Chapter decided to redecorate the whole main chapel in a baroque style. The placing of marbles and baroque adornments made the paintings at the apse disappear, but frescoes of the domed vault were covered by a new one placed 80 cm below, which preserved the paintings up to date.

The existence of these renaissance frescoes was documented in the cathedral archives, but it was thought that paintings were in a poor condition. In June 2004, during restoration work on the main chapel, it was a surprise to discover that the preservation state of these frescoes was remarkable, apart from the extraordinary beauty of this work of art. The Chapter requested their restoration, and to access them it was necessary to remove all severies of the baroque dome. The restoration works finished in December 2006. A photo gallery illustrating the renaissance frescoes, their discovery and restoration process is accessible at the cathedral’s website (http://www.catedraldevalencia.es).

Given that some salt efflorescence was found during the restoration process [19], the roof above the apse was remodeled to avoid infiltration of rainwater through it. Roof tiles are effective in carrying rainwater away, but it was decided to remove them in order to reconstruct the terrace as it was in the original gothic construction.

**Figure 1.**
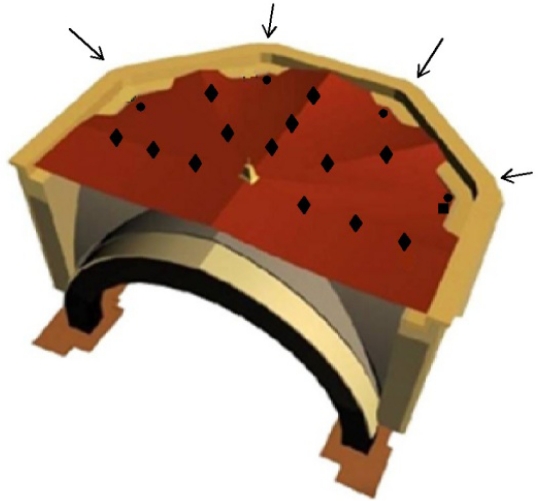
Perspective of the upper part of the apse and terrace above the frescoes. The four arrows indicate the position of storm drains. Six sensors are located at the terrace: four touch-switch sensors activated in case of flooding by obstruction of the drainage channel (circles) and one rain-activated touch-switch sensor (square). The position of ventilation tubes is also indicated (diamonds).

The new terrace is composed of flat tiles (30 × 15 cm) made of baked clay. They are laid on different planes whose intersections converge to the position of the vault keystone (Figure 1). The disposition of these planes, which resembles a hand-held folding fan, provides an effective evacuation of rainwater towards four storm drains located at the terrace borders. These drains evacuate the rainwater through four waterspouts with a quadratic section of about 150 cm^2^ (Figure 1, arrows). Pigeons sometimes take refuge inside these drainage channels, which increases the risk of objects that might hinder the drainage. Taking into account this potential risk, the restoring team decided to install four touch-switch sensors that would be activated as an alarm mechanism if one of these waterspouts is obstructed and part of the terrace gets flooded.

During the terrace remodeling, an asphaltic roofing sheet was laid under the flat tiles. These sheets are commonly used for waterproofing of terraces, and a test is often conducted by flooding them and checking signs of infiltration. But such test was not carried out in this case to avoid unnecessary risks on the frescoes. Laying this asphaltic sheet was more complicated than in a conventional terrace because it had to be sealed to many encounters: terrace borders, storm drains and waterspouts. Additionally, the asphaltic sheet had to be sealed to 13 ventilation tubes that cross vertically through the terrace down to the frescoes and communicate the indoor and outdoor environments (Figure 1, diamonds). These tubes are properly covered to prevent the entrance of rainwater. Given that flat tiles and the mortar between them (1 cm of width) are porous materials that absorb water by capillarity, the presence of any joint not properly sealed or any crack in the sheet might represent a source of water infiltration. In our opinion, this risk is not negligible and it will increase along the decades. It is very difficult to ensure a perfect impermeability of the terrace over the frescoes, and hence some kind of maintenance or periodic control should be conducted given the value of such a piece of art that is to be conserved over centuries.

The distance from frescoes to the terrace above them is about 50 cm next to the vault keystone, but it increases towards the terrace borders. This space between the severies and the terrace is filled with lime-sand materials that are rather permeable. The probability of rainwater infiltration through the terrace is small, but if it occurs, water will be absorbed by the filling material. Given that the asphaltic sheet prevents water evaporation through the terrace, small amounts of water accumulated year after year would diffuse by capillarity throughout the filling material down to the frescoes and might reach the paintings and cause humidity problems.

The importance of waterproofing in the roof above frescoes is discussed in the literature. St. Stephan’s church in Nessebar, Bulgaria, was restored in 1975, but severe deterioration processes of the mural paintings were observed after some years. The investigations revealed great errors in design and construction of the waterproofing and drainage system. Roof tiles were repaired to stop all means of external water penetration as well as to prevent humidity dropping down through capillary action. These modifications were effective in rectifying the situation [20].

During more than 300 years, the renaissance frescoes of the Valencian cathedral have remained under rather stable conditions of temperature, *RH* and light. But this is not the case from now on. In order to assess the factors involved in their long-term conservation, it was decided to implement a monitoring system comprised by temperature and *RH* sensors, which were installed during the restoration process at different points of the vault as well as on the paintings surface as described elsewhere [21]. This control system is rather unique because sensors are rarely inserted into the precious walls supporting the frescoes.

Monitoring temperature and *RH* was intended for several purposes. On one hand, the detection of humidity increments at specific parts of the vault might indicate infiltration of water through the roof, which would require corrective measures as in the case described by [20]. On the other hand, recordings of excessive diurnal or seasonal thermo-hygrometric variations would lead to corrective recommendations. The present work reports the analysis of *RH* data obtained during the first four years of monitoring and highlights the advantages of humidity sensors inserted in frescoes for preventive conservation.

The Italian Standard UNI 10829 (1999) defining monitoring, elaboration and analysis of the microclimatic data as supporting actions for artefacts preservation, led to the need of a long-term monitoring and of a statistical approach for the data management. The approach proposed by the Italian Standard has been recently adopted by the European Standard EN 15251 (2007) [22]. In accordance with these standards, a statistical procedure is applied in this paper to assess the long-term thermo-hygrometric microclimate of fresco paintings that was firstly proposed in [21].

## 2. Materials and Methods

### 2.1. Description of Probes: Characteristics and Installation

Four touch-switch sensors (Figure 1, circles) were located at the storm drains that evacuate rainwater falling over the terrace above the frescoes. These sensors are activated if the water level in the drainage channel is too high, which would occur only if the channel is obstructed. In that case, an immediate actuation will be required due to the disastrous consequences that might be derived from a flooding. Up to date, these sensors have never been activated (*i.e.*, a constant value was recorded all the time). There is an additional touch-switch sensor of the same type, also located outside the cathedral, which is activated by rainwater.

**Figure 2.**
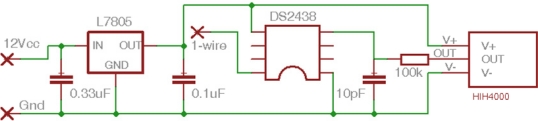
Electronic scheme of probes for measuring *RH* and temperature: voltage regulator L7805, *RH* sensor (HIH-4000) and integrated circuit DS2438. The latter incorporates a temperature sensor and one A/D converter.

The indoor microclimate is monitored by means of 29 probes: seven of them are located on the vault ribs, two at the apse cornice, 10 on the walls and 10 probes are installed on the surface of frescoes. An additional one is outside the cathedral, but a preliminary study revealed that this probe was broken and it was disregarded. Each probe (Figure 2) contains an integrated circuitry humidity sensor (HIH-4000, Honeywell International, Inc.) with an accuracy of ±3.5%*RH* as well as a small-outline integrated circuit (SOIC), model DS2438 (Maxim Integrated Products, Inc.) that incorporates a temperature sensor with an accuracy of ±2 °C. This SOIC is designed for on-chip measurement of battery temperature and voltage. Probes were calibrated prior to their installation as described in a previous study [21].

**Figure 3.**
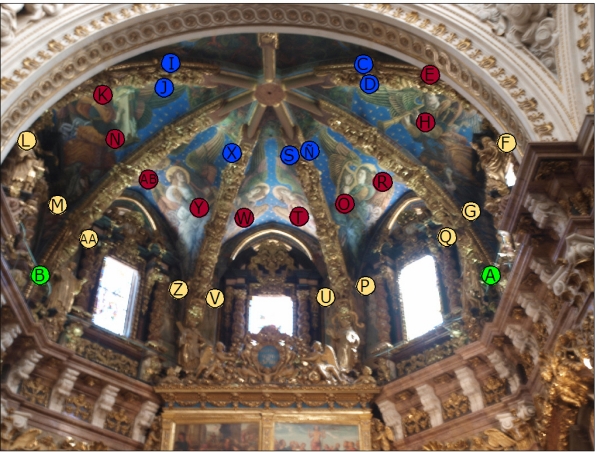
Renaissance frescos at the cathedral of Valencia decorating the vault severies above the presbytery. The seven ribs converge to the vault keystone. The position of 29 probes installed in 2006 for indoor air monitoring are indicated in colored circles: two of them at the cornice (green), seven probes on the ribs (blue), 10 on the walls below the severies (yellow) and 10 on the frescoes (red).

**Figure 4.**
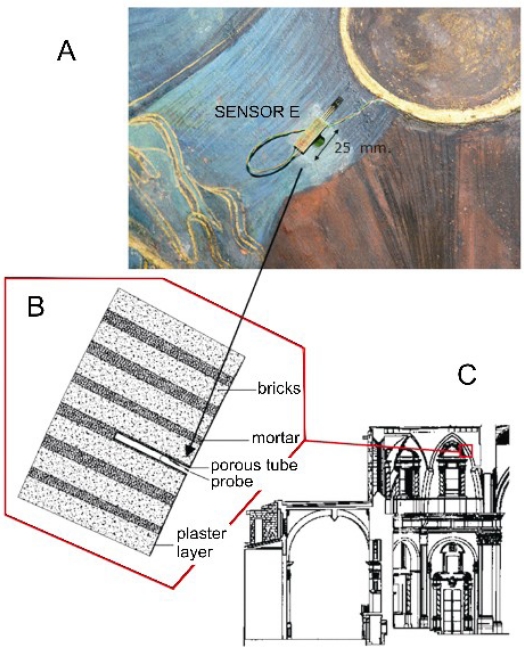
Installation details of probes for the microclimate monitoring of fresco paintings. (**A)** picture showing Sensor E (*RH* and temperature sensors assembled as indicated in Figure 2) and the hole (opening of the ceramic tube) where it was inserted in reintegrated plaster layer. Wires were properly dissimulated by the restoring team as shown in this picture. (**B**) The tube was introduced between two bricks. (**C**) vault roof supporting the frescoes.

The installation of probes was as following. (i) One probe was located on each rib separating two vault severies (Figure 3, blue circles), at about 2 m from the keystone and 17.5 m from the floor. These probes as well as their respective wires are not visible from beneath because they are hidden by the baroque adornments of ribs, which were left when the baroque dome was removed. These sensors measure the air temperature and *RH* at about 5 cm from the surface of frescoes. (ii) Two additional probes are located at the apse cornice, at 13 m from the floor (Figure 3, green circles). (iii) Ten probes are located on the apse walls below the severies, at a distance of about 120 cm from the severy, 20 cm over the upper part of the stained glass windows (Figure 3, yellow circles) and at a height of 16 m from the floor. These windows are designed for lighting and do not allow air exchange with the outer environment. A hole of 18 mm was drilled in the mortar between two ashlars, and a white porous ceramic tube made of baked white clay (160 mm long, 16 mm of outer diameter and 12 mm of inner diameter) was inserted into the wall. The exact position of bricks and joints was identified by means of an ultrasound device (a laboratory prototype) as described elsewhere [23]. After introducing the tube into the hole, it was fixed using a sand-lime mortar. One probe was located inside each tube (Figure 4) so that the *RH* sensor remains on the wall surface. This sensor is therefore supposed to record the air *RH* at the boundary layer in contact with the wall. The *RH* can be different if measured some centimeters away from the wall, because transport mechanisms of water between the air and the limestone wall, related with water adsorption/desorption dynamics, might occur at the boundary layer. The temperature sensor remains about 3 cm inside the wall and it measures the air temperature inside the tube. (iv) Ten porous tubes of the same type as (iii) were also inserted in the frescoes by drilling in the mortar (15–20 mm of thickness) between two bricks of the severy (Figure 3, red circles) at a height of about 17 m from the floor.

It is obviously nonsense to drill the original fresco painting of such a valuable work of art, given that different studies mentioned in the introduction have shown that it is possible to assess the potential risks involved in the conservation of frescoes by microclimate monitoring without the need of inserting probes into the precious walls. Nevertheless, given that the restoring specialists had to remove and reintegrate the plaster layer supporting the paintings in zones highly deteriorated due to humidity problems, it was decided to install the probes in such zones (Figure 4). These probes are supposed to provide information about the conservation state of paintings. Thus, if any of them records abnormally high *RH* values that turn out to be clearly different from the *RH* measured at other parts of the vault, this result might indicate problems of water infiltration through the terrace.

### 2.2. Data Acquisition System

Three electric wires come out from each probe: one for 12 V DC power supply, one for 1-wire data transfer and one wire for ground. All cables were properly dissimulated by the restoration team using aquarelle painting according to the colors, drawings and patterns of the paintings behind (Figure 4), so that it is difficult to distinguish the presence of wires even at few meters of distance.

**Figure 5.**
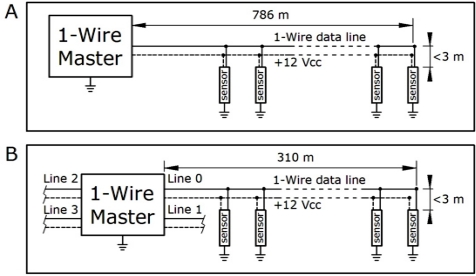
Scheme indicating the parallel configuration of sensor data recording. (**A**) initial configuration used until December 2008 based on a single bidirectional data line. (**B**) new configuration comprised by 4 data lines used from January 2010.

According to the recommendations of the 1-wire manufacturer [24] the installation was carried out using 4-pair Ethernet copper plaited wires (category 5 cable) with a diameter of 0.5 mm, based on a linear wire configuration [Figure 5(A)], which is supposed to be more robust.

Once the cable installation was finished, the total electric resistance of the 1-wire data line was measured for estimating its total length. Taking into account that the reading obtained from each *RH* sensor is affected by the incoming voltage, this value was accurately measured in all *RH* sensors once finished their installation in 2006. It ranged from 4.964 to 5.006 V. The variability of incoming voltage was taken into account to make the appropriate corrections in transforming the sensor readings into *RH* values, using the formula suggested by the manufacturer (http://sccatalog.honeywell.com/pdbdownload/images/hih-4000.series.chart.1.pdf). For sensors A, B, L, G and W, it was checked that their incoming voltage remained constant along the 4 years of data recording. Unfortunately, this check was not possible for the remaining sensors as it would require the use of scaffolding.

Sensor data were recorded by means of a 8051 microcontroller connected through a 1-wire bus with all probes. The 1-wire bus is designed for the communication between a master microcontroller and a slave located relatively close, though in practice the cable can be quite long. In order to achieve an effective communication with probes, an advanced 1-wire network driver was used [25,26].

The 8051 microcontroller was programmed in assembler language. The communication with all probes resulted appropriate, despite the fact that the 500 m of maximum cable length recommended by the manufacturer was exceeded considerably [25]. Actually, the total length of the wire installed in the vault was about 786 m [Figure 5(A)]. The length of secondary cable connecting each probe to the bidirectional 1-wire data line was lower than 3 m in all cases (Figure 5).

Despite the appropriate performance of the sensor-microcontroller communication system, the setup was complicated because each software modification, which was frequent at the beginning, required the intervention of a specialist in assembler language. Moreover, the system had a rather limited data storage capacity. Data were saved in the 8051 microcontroller memory, which was able to keep one datum from all sensors recorded every hour along two weeks. Thus, it was necessary to download the recordings twice a month. Data were collected with this system during February and the last quarter of 2007.

Given the inconvenience of having to access every two weeks the vault cornice where the microcontroller was located, in January 2008 it was decided to communicate the 8051 microcontroller with a PC under a LabVIEW (http://www.ni.com/labview) environment in order to save the data in the hard disk. The data acquisition frequency was increased to one recording per minute because the PC memory was able to store nearly an unlimited data amount. This system presented new problems: (i) the computer had to be connected permanently and (ii) the PC operative system crashed from time to time due to unknown causes. Because of this, data from several periods of 2008 were lost, and it was necessary to check periodically that the PC was working correctly.

The microcontroller broke down in December 2008 and, due to the mentioned problems with the PC, it was decided to replace the data acquisition system by a new one, which was operative in January 2010. The electronics prototyping platform Arduino (http://www.arduino.cc) was selected for the new system because it is an open-source flexible microcontroller, easier to be programmed and with an altruistic web community devoted to provide and spread programming examples. The driver DS2482-800 was chosen to manage the bus. This chip transforms the communication protocol I2C (easily generated by the Arduino through its Wire library) into a 1-Wire protocol.

One drawback of this system is that it was unable to communicate with all slave probes if they were connected to the same line. Taking into account that this chip can manage up to eight different lines, the original set up was divided into four lines with the same parallel configuration [Figure 5(B)]. The longest line of the new configuration was 310 m of plaited wire, which exceeded again more than 50% the maximum length recommended by the manufacturer that was about 200 m for the chip employed.

Instead of using a PC, data were saved in a 4 GB USB memory by means of a VDIP1 modulus from Future Technology Devices Intl. Ltd. This modulus comprises a microcontroller that manages the protocol to open, close, save, create or delete files by using short commands similar to those of MS-DOS. These commands are transmitted from the Arduino platform to this modulus through the series port of the microcontroller.

Apart from not requiring a PC for data storage, one advantage of this new system is that it was programmed to record the incoming voltage of each sensor before its output reading, which allows *RH* measurements more reliable at the long term. This system started collecting data in February 2010.

### 2.3. Multivariate Data Analysis

*RH* data recorded in 2007 were arranged in a matrix comprised by 432 observations (time instants, in rows) by 25 variables (*RH* sensors, in columns). This matrix was row-centered as described in [21]. Next, a principal components analysis (PCA) was carried out using the software SIMCA-P 10.0 (http://www.umetrics.com). The same analysis was repeated with sensor data recorded in 2008 with 409,312 observations and 2010 with 429,012 observations. Results from these three models were compared in order to check if the relationships among sensors were maintained year after year. Moreover, an interpretation was provided for the first principal component (PC1) and the second principal component (PC2), which account for the relevant data variability. Data of temperature recorded in 2008 and 2010 were also analyzed using PCA.

### 2.4. Recalibration of Sensors

In order to check if sensors work properly after the four years of operation, it was decided to conduct a recalibration. Unfortunately, it was only possible to access five of the 29 sensors (A, B, L, G and W), which were removed and uninstalled from their original position in August 2011. These sensors were introduced in a climatic chamber model alpha 990-40H from Design Environmental Ltd. (Gwent, UK). Additionally, four sensors of the same type calibrated by the manufacturer in 2011 were also introduced. The temperature was maintained at 20 °C. The *RH* was set initially at 15% and was increased progressively up to 80% in nine steps, each one lasting 2 h. Thus, the total duration of the experiment was 18 h. The data acquisition rate (T^a^ and *RH*) was one datum per minute from each sensor. Next, the average of all data recorded by each sensor in each step was calculated in order to obtain the bias of sensors uninstalled from frescoes with respect to those calibrated by the manufacturer. Sensors A, B, L, G and W were reinstalled after the recalibration. It was discovered that sensor G was not operative because one terminal was wrongly welted and it was repaired.

## 3. Results and Discussion

### 3.1. Data Available from the Monitoring System

The thermo-hygrometric monitoring system was set up in February 2007 with a frequency of one datum recorded per hour. *RH* data collected in the first year of operation (February, September, October and November 2007) were analyzed in a recent work [21]. At present, the following data periods are also available: from mid-January 2008 to mid-December 2008, and from mid-February 2010 to 31 December 2010. Table 1 lists all sensors of T^a^ and *RH* with a correct operation and time periods that will be analyzed in the present work. Data collected in 2011 will be used in future studies. The communication between each sensor and the microcontroller was guaranteed with cyclic redundancy checks that were implemented in our software. Non-operative sensors indicated in Table 1 were those that failed in the communication with the microcontroller.

Given that the main target of the monitoring system is to detect humidity problems in the frescoes, it can be assumed that *RH* recordings will provide much more information for this purpose than temperature measurements. Taking into account that the manufacturer recommends a correction of *RH* estimations according to temperature (http://sccatalog.honeywell.com/pdbdownload/images/hih-4000.series.chart.1.pdf), the proposed correction was applied in each observation in order to improve the accuracy.

**Figure 6.**
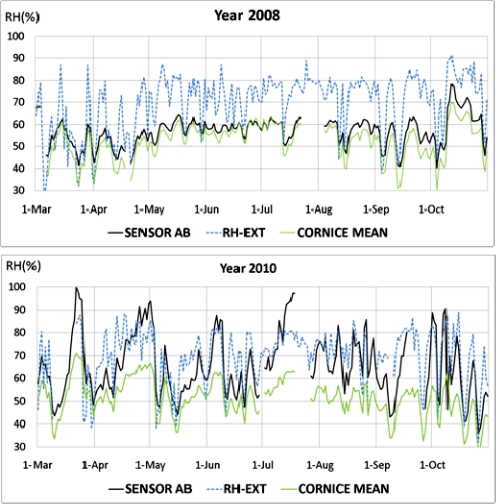
Evolution of *RH* data recorded in 2008 and 2010 by sensor AB (black line) and those at the cornice (mean of sensors A and B). Measurements outside the cathedral provided by a nearby weather station are also shown (dashed blue line).

**Figure 7.**
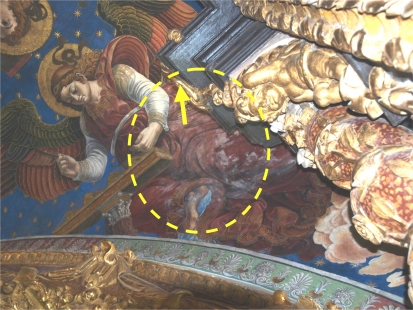
Picture of the fresco painting where sensor AB is located (exact position indicated with an arrow). The dashed ellipse highlights a zone with white salt efflorescence stain. The picture was taken in March 2011.

Data from sensor AB recorded in 2010 were found to be abnormal in the multivariate statistical analysis for *RH* as described below. Figure 6 shows that sensor AB presented in 2010 values similar to the outside *RH*, but in 2008 the recorded values were similar to measurements at the cornice. The trajectory of AB with respect to cornice sensors in 2010, which are not parallel, discards a pattern caused by an aging drift of sensor AB [27]. Consequently, it was discarded for the statistical analysis. Although it is unknown why this sensor performs differently from the rest of painting sensors, it is noteworthy that formation of salt efflorescence can be observed in the zone where this sensor is located (Figure 7). Thus, the different pattern of sensor AB might be caused by an abnormal humidity problem in the frescoes.

**Figure 8.**
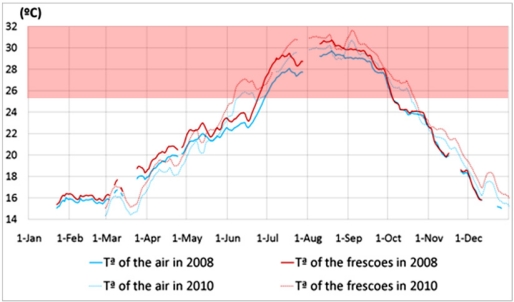
Evolution of *T^a^* data recorded in 2008 (solid lines) and 2010 (dashed lines) by sensors measuring the air (mean of A and B) and temperature of the paintings sensors (see Table 1) that were operative in each year.

Saline efflorescences are caused by water movements diffusing thorough the roof or by condensation due to a lower temperature of the roof with respect to the surrounding air [28]. The latter situation is rather unlikely because roof temperature was always slightly higher than the air (Figure 8). Even if atmospheric pollution could be an important source of salt ions, it must not necessarily be considered the main source inside monuments [29]. Air pollution in the Cathedral of Valencia is rather limited due to the low presence of visitors and its good natural ventilation along all the year. Moreover, the use of light candles and incense is restricted to few days in the year. According to a preliminary study [30], salts found during the restoration of these frescoes in 2007 were nitrates and sulphates that could come from the stone ashlars, original mortar or the Portland cement used in recent restorations of the terrace. Previous studies indicate that most nitrate efflorescences are formed when the air *RH* is lower than 60% at 25 °C, while sulphate efflorescences precipitate with air *RH* below 88% [29]. This fact, together with the abrupt drops of *RH* in Valencia, can produce salt precipitations caused by water diffusion through the limestone ashlars of the apse, which are very porous.

The Spanish Ministry of Science and Innovation has recently funded a research project to further study the preventive conservation of frescoes. In the framework of this project, a scaffold will be put to access the paintings, which will allow (i) to study why sensors H, U and Y were operative up to 2008 but not in 2010, (ii) to replace sensors F, M, and N that were never operative by new ones, and (iii) to check if sensor AB works correctly, which would confirm that its higher measurements are likely to reflect humidity problems.

Outside *RH* measurements with an accuracy of ±3% were provided by the Environmental Service of our university (Universidad Politécnica de Valencia), and were collected by a meteorological station located 4 km East of the cathedral. Figure 6 shows that sensors A and B at the cornice follow the same cycles as *RH* measurements outside the cathedral but with a lower amplitude, which suggests that the sensors accuracy is adequate. This buffered pattern of indoor sensors is caused by the reduced ventilation inside the cathedral and the microclimate protection created by the thick walls of the cathedral.

Figure 6 also reflects that the pattern of AB in 2010 differs from the signal shape of sensors at the apse cornice as well as from outdoor *RH* measurements. Actually, when the outer *RH* is low, it takes values similar to sensors A or B, but during the periods of high *RH* (e.g., April 2010), the recordings of AB tend to increase progressively. Given this unique performance and taking into account the presence of efflorescence in the vicinity, it seems that this sensor works correctly and is actually reflecting humidity problems in the frescoes. The fact that AB behaved as other sensors in 2007 and 2008 but not in 2010 (Figure 6) is an important issue that encourages further monitoring in the next years. If such humidity problem has been detected by the system in just 4 years, what corrective and preventive measures should be adopted for the conservation of the precious frescoes in the long term? This question is out of the scope of the present work but should be tackled by experts in the field.

The Italian Standard UNI 10829 (1999) establishes that the range of acceptable temperature for the conservation of frescoes is 6–25 °C while in the case of *RH*, this range is 45–60%. The maximum hourly variations of temperature and *RH* in the case of plaster are 1.5 °C/h and 2%/h, respectively, but these values are not documented for fresco paintings. Nonetheless, variations higher than these recommendations for plaster were not found in the recordings from frescoes monitored in this study.

Figure 8 shows that the average temperature was similar in 2008 and 2010. Moreover, the air temperature surrounding the frescoes was slightly higher than the inside air in the cathedral. Temperatures above 25 °C are highlighted in the Figure 8 because they are not in the range recommended by UNI 10829 (1999). Temperatures below 6 °C were never recorded by the monitoring system.

The UNI 10829 and the EN 15251 standards provide recommendations for temperature and *RH* in preventive conservation of frescoes. Table 2 indicates the number of days in 2008 and 2010 when the temperature or *RH* were out of the recommended range. During many days along the year, the average temperature or *RH* is out of the recommended range, which might seem a risk for the conservation of frescoes. However, it is important to take into account that both standards were developed for the conservation of artefacts in museums, which require in general rather stable conditions that are often difficult to achieve inside churches. In such cases, it is necessary to monitor year after year the mean microclimatic parameters of the painting in order to find if they have changed.

**Figure 9.**
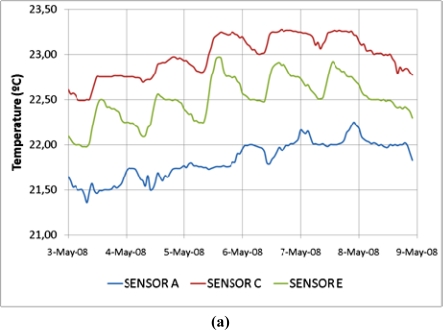

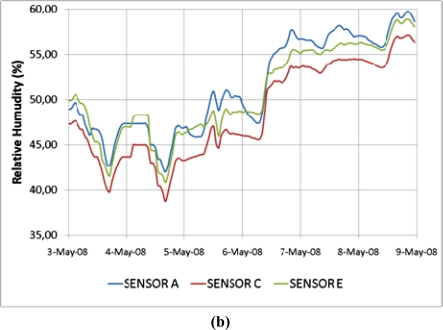
Evolution of T^a^ **(a)** and *RH* **(b)** for three sensors measured from 3 to 8 May 2008.

Although the measurement error of the thermometer is ±2 °C according to the manufacturer, Figure 9 suggests that the actual error working with averages is much lower, as sensors C and E are clearly reflecting daily variations of temperature in a range of about 0.4 °C. This graph has been made with the hourly mean taken at a frequency of one datum per minute.

### 3.2. Statistical Study of Temperatures

Temperature data recorded in 2008 were analyzed using PCA. It was found that PC2 reflected a different performance in winter *versus* summer. In particular, two shifts that occurred on 1 June and 3 November suggest a change in the management of the natural ventilation inside the cathedral. Sensors that most determine PC2 are V, Q and M. For some reason, these three sensors installed in walls have changed, so that their values in winter are above the mean trajectory, while in summer they remain below the mean.

If the same multivariate analysis is repeated with data recorded in winter 2008, another shift is detected in PC2 on 25th February. Sensors A and B are the ones responsible for this shift as well as those located on ribs (*i.e.*, all sensors measuring the inner air temperature). It seems that some kind of operational change occurred in the ventilation management on this date. This change was detected by sensors measuring air temperature but not by those installed on the walls or paintings, whose temperature variations are more buffered. PC2 provides useful information to diagnose the effect of operational changes in the ventilation management. The natural ventilation of the cathedral is controlled by the opening and closing of doors and certain windows, which is not properly recorded.

**Figure 10.**
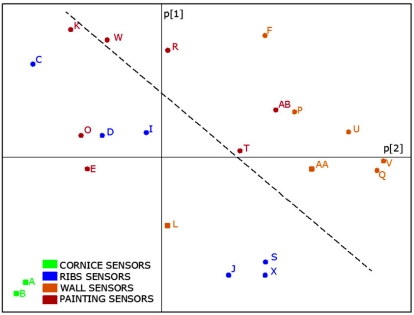
Loading plot corresponding to PC1 and PC2 (*i.e.*, p[1] *versus* p[2]). This plot was obtained by applying PCA to T^a^ data recorded in 2010. The matrix was row-centered prior to the PCA.

**Figure 11.**
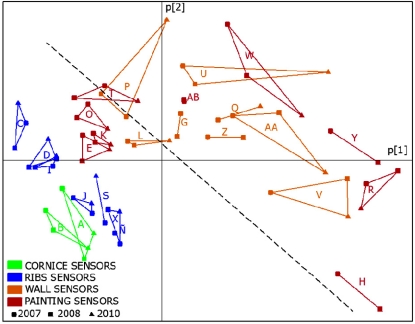
Overlap of three loading plots corresponding to PC1 and PC2 (*i.e.*, p[2] *versus* p[1]). Each plot was obtained by applying PCA to *RH* data recorded in a different period: 2007 (circles); 2008 (squares); 2010 (triangles). In all cases, matrices were row-centered prior to the PCA as explained elsewhere [21]. Points joined together correspond to the same sensor (labels as in Figure 3).

Another PCA was fitted for temperature data recorded in 2010. In this case, no sudden shifts are detected in PC2, which seems more reasonable than in the 2008 data analysis. The loading plot for PC1 and PC2 (Figure 10) presents certain similarity to the corresponding loading plot obtained with HR data (Figure 11). Actually, sensors J, S and X are close to each other in both plots, as well as sensors C, D and I. In order to better compare both plots, a dashed line was inserted in Figures 10 and 11. Interestingly, all sensors appearing to the left of this line in Figure 10 are also located to the left of the line in Figure 11. This result suggests that PC2 of temperature recordings also provide relevant information related with *RH* measurements. Nonetheless, this conclusion is not supported by 2008 temperature data, as discussed above, and further work should be conducted in the following years to better investigate this issue.

Given that a clear shift was detected in 2008 on 1 June and 3 November, the data series was divided into three periods: (1) data recorded up to 10 May, (2) from 10 May to 29 September and (3) data recorded from 29 September until 31 December. For each period, the average temperature recorded by each sensor was calculated, for the years 2008 and 2010.

The correlation between the average temperature recorded by each sensor in 2008 was calculated, as well as for the year 2010 is statistically significant (r = 0.603, p = 0.005). Sensors R and W appear as outliers. If both are discarded in the analysis, the correlation increases (r = 0.813, p < 0.0001).

Sensors R and W also appear as outliers if the regression analysis is conducted with data of period 1. After discarding both sensors, a similar correlation is obtained (r = 0.820, p < 0.001).

In the case of period 2, no outliers appear but the correlation is lower (r = 0.559, p = 0.01). Unexpectedly, the correlation for data in period 3 is not statistically significant (p > 0.05).

The reason for the abnormal performance of sensors R and W with respect to their average temperatures is unknown. It could be caused by the replacement of a window at the center of the apse vault carried out in 2009. This window did not allow ventilation when closed, and it was left open or close randomly. It was replaced by a new window that allowed ventilation even when it was closed. However, this is just a hypothesis and further analyses of data recorded in the next years should be carried out.

### 3.3. Comparison of RH Data Recorded in 2007, 2008 and 2010

An important objective of the present work was to investigate if the monitoring system worked similarly in the years 2007, 2008 and 2010. Taking into account that data were acquired every hour in 2007 but every minute afterwards, one option is to carry out PCA models with all available data in 2008 and 2010, but another option is to work with hourly average data. Nevertheless, very similar results would be obtained in both cases because *RH* values recorded in one minute are very similar to those measured in the following minutes, and the presence of “redundant” data is not a problem for PCA. It was decided to work with hourly average *RH* in 2008 and 2010 because the amount of data is more handy, particularly with a future perspective. One option for the monitoring system would be to record one datum per minute, compute the average every hour and save only these averages, which would turn into a practical and efficient acquisition system.

Three PCA models were obtained following the same methodology described in the previous study [21]: one model with all available data from 2007; another with data of 2008 and a third one with data recorded in 2010. These analyses were carried out with data from operative sensors indicated in Table 1. As stated above, AB was discarded given its particular performance. The comparison of results will allow us to discuss if the relationships among sensors (*i.e.*, similarities and dissimilarities) have been maintained along the years under study. From each one of these models, we obtained the loadings of variables (sensors) in the formation of PC1 and PC2, that will be referred to as p[1] loadings and p[2] loadings respectively. Next, the three loading plots were overlaid (Figure 11).

Figure 11 shows that relationships among sensors are basically maintained, which implies that the monitoring system works correctly despite the change in the data acquisition system carried out in 2009. These consistent results, as well as the ones reported in a recent study [31] highlight that PCA is a powerful statistical tool to characterize the different performance among sensors of the same type located at different positions. Many applications showing the advantages of PCA for sensor diagnosis have been reported [32,33].

### 3.4. Interpretation of RH Relevant Principal Components

Given that the monitoring system is intended to operate for years, it is relevant to discuss the convenience of working with data averaged hourly or even daily. In order to investigate this issue, one PCA was conducted with data collected along 2008 with a frequency rate of one datum per minute. Next, the study was repeated with daily averages. The results were very similar, as the correlation was very high (r > 0.9999) between p[1] loadings from both models as well as in the case of p[2]. The same study was repeated with 2010 data and similar results were obtained. This is of interest because it simplifies the data storage in long-term monitoring systems with many sensors. Our study continues with the PCA results using daily averages, as the purpose is to obtain a methodology not requiring an excessive data amount.

#### 3.4.1. Study of the First Principal Component

**Figure 12.**
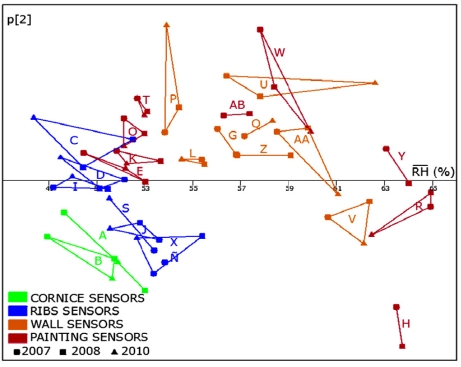
Scatterplot of p[2] (same values as Figure 11) *versus* the average *RH* data recorded by each sensor in one year (2007: circles; 2008: squares; 2010: triangles). Points joined together correspond to the same sensor.

In our previous work [21], it was stated that the p[1] loadings were correlated with the average *RH* signal. In order to further study this issue, we averaged all *RH* data recorded from each sensor along 2008 and 2010, which was called ${\bar{\mathrm{RH}}}_{2008}$ and ${\bar{\mathrm{RH}}}_{2010}$, respectively. The correlation between ${\bar{\mathrm{RH}}}_{2008}$ and p[1]_2008_ is statistically significant (r = 0.997, p < 0.0001), as well as between ${\bar{\mathrm{RH}}}_{2010}$ and p[1]_2010_ (r = 0.994, p < 0.0001). Given this high correlation, it is possible to replace p[1] values in Figure 11 (horizontal axis) by the average *RH*, as shown in Figure 12. Both plots are obviously very similar, but using average values instead of p[1] seems more convenient for an easier interpretation.

#### 3.4.2. Study of the Second Principal Component

In the reported study of 2007 data [21], it was also stated that PC2 discriminated sensors according to the different signal shape. It would be of interest to characterize these differences and find a physical meaning for PC2. For this purpose, different parameters described next (HMV, DMV, MMV) were calculated to characterize the pattern of *RH* (*i.e.*, signal shape) *versus* time.

The hourly average *RH* is calculated as the average *RH* recorded in 1 h [Equation (1)], being *RH*_min_ the value acquired every minute by the sensor:

(1) $${\bar{\mathrm{RH}}}_{h}=\frac{\sum_{\text{min}=0}^{\text{min}=59}{\mathrm{RH}}_{\text{min}}}{60}$$

The hourly mean variation during a time period of H hours is calculated as the mean of absolute differences between two consecutive hourly *RH* averages [Equation (2)]. For example, if data corresponding to 7,200 h have been collected in one year: H = 7,200; *RH_1_* = average *RH* recorded in the first hour; ${\bar{\mathrm{RH}}}_{h}$ average *RH* recorded in the h-th hour. The absolute value is used to obtain the variation between one hour and the previous one, no matter if this difference is positive or negative. This calculation is equivalent to compute with ${\bar{\mathrm{RH}}}_{h}$ values the average of moving ranges with order 2:

(2) $$\mathrm{HMV}=\frac{\sum_{h=2}^{H}\left|{\bar{\mathrm{RH}}}_{h}-{\bar{\mathrm{RH}}}_{h-1}\right|}{H-1}$$

The daily mean variation was calculated in the same way, as the mean of absolute differences between two consecutive daily *RH* averages, being D the total number of days (Equation 4):

(3) $${\bar{\mathrm{RH}}}_{d}=\frac{\sum_{h=0}^{h=23}{\bar{\mathrm{RH}}}_{h}}{24}$$

(4) $$\mathrm{DMV}=\frac{\sum_{d=2}^{D}\left|{\bar{\mathrm{RH}}}_{d}-{\bar{\mathrm{RH}}}_{d-1}\right|}{D-1}$$

Similarly, the monthly mean variation for a period of M months, assuming 30 days per month, was obtained according to Equation (6):

(5) $${\bar{\mathrm{RH}}}_{m}=\frac{\sum_{d=1}^{d=3D}{\bar{\mathrm{RH}}}_{d}}{30}$$

(6) $$\mathrm{MMV}=\frac{\sum_{m=2}^{M}\left|{\bar{\mathrm{RH}}}_{m}-{\bar{\mathrm{RH}}}_{m-1}\right|}{M-1}$$

Next, a multiple linear regression was fitted with the software Statgraphics 5.1 to study if p[2] values could be predicted as a function of these $\bar{\mathrm{RH}}$ averages and mean variations. After trying several models with different combinations of predictive variables, the best results were obtained with yearly *RH* average $\left(\bar{\mathrm{RH}}\right)$ and daily mean variation (DMV). Equations (7) and (8) are the resulting regression models fitted for the p[2] values corresponding to 2008 and 2010, respectively:

(7) $$p\left[2\right]=232.88-2.695\cdot \bar{\mathrm{RH}}-32.395\cdot \mathrm{DMV}$$

(8) $$p\left[2\right]=297.03-3.869\cdot \bar{\mathrm{RH}}-32.122\cdot \mathrm{DMV}$$

**Figure 13.**
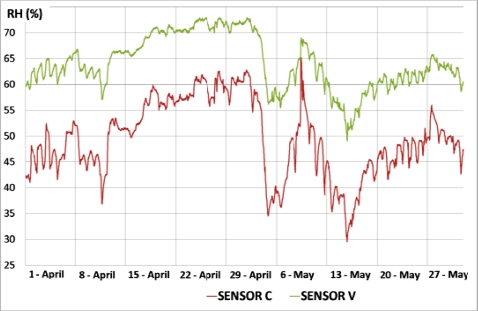
Evolution of *RH versus* time for sensors C and V (data recorded in April and May 2010).

In both equations, regression coefficients associated to $\bar{\mathrm{RH}}$ are statistically significant (p < 0.0001) as well as coefficients associated to DMV (p < 0.0001). Given that Equations (7) and (8) are rather similar, it would be of interest to obtain a consensus model for the prediction of p[2] in both years. If a new joint regression model is fitted using an indicator variable to study the effect of year (*i.e.*, 2008 *versus* 2010 data) and considering a possible interaction with the two variables included in the model, it turns out unexpectedly that Equations (7) and (8) cannot be condensed into a unique predictive model. The reason is uncertain, and further data will be necessary to study this issue with more detail. Taking into account that the determination coefficient of Equation (8), R^2^ = 0.942, is higher than in the case of Equation (7) (R^2^ = 0.839), it was decided to adopt for the prediction of p[2] in both years as a function of two parameters with physical meaning extracted from sensor data: the average *RH* and daily mean variation. In Equation (8), DMV is more important than $\bar{\mathrm{RH}}$ as the former yields a higher correlation with p[2] (R^2^ = 0.734) which implies that, roughly speaking, PC2 discriminates the sensor performance according to daily mean variations. Parameters $\bar{\mathrm{RH}}$ and DMV are negatively correlated, which is consistent with Figure 13. Actually, daily *RH* variations (*i.e.*, Higher DMV values) are observed for the sensors with lowest *RH* averages.

According to Figure 12, one of the lowest average *RH* recordings corresponds to sensor C, and the opposite applies to V. Figure 13 shows the *RH* recordings *versus* time for both sensors. It can be observed that the amplitude of daily cycles is clearly higher in sensor C, which implies a higher value of DMV. Taking into account that sensor C records lower *RH* values in average, this result suggests a negative correlation between DMV and *RH*. Actually, this correlation is statistically significant (r = −0.74, p = 0.0001) using data of the year 2008.

**Figure 14.**
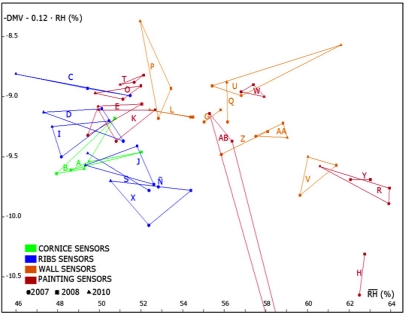
Plot of $\left(-\mathrm{DMV}-0.12\cdot \bar{\mathrm{RH}}\right)$ *versus* the average *RH* data recorded from each sensor in one year (2007: circles; 2008: squares; 2010: triangles). Points joined together correspond to the same sensor. Point 2010 of sensor AB is out of the graph.

In summary, PC1 can be interpreted as the yearly *RH* average while PC2 provides information basically about daily mean variations. Equations (8) and (9) are equivalent after rearranging the terms, though the latter provides an easier physical interpretation. Thus, as $\bar{\mathrm{RH}}$ and DMV have physical meaning, the parameter $\left(-\mathrm{DMV}-0.12\cdot \bar{\mathrm{RH}}\right)$ could be used instead of p[2] in Figure 12. The resulting plot (Figure 14) is quite similar to Figures 11 and 12:

(9) $$0.0311\cdot p\left[2\right]-9.2469=-\mathrm{DMV}-0.12\cdot \bar{\mathrm{RH}}$$

This type of plot will be very useful to study and diagnose the evolution year after year of data collected from sensors. Those ones close to each other in Figure 11 will present a similar performance (*i.e.*, similar average values and *RH* pattern with a similar shape).

#### 3.4.3. Study of RH Further Principal Components

Apart from PC1 and PC2, it is of interest to study if PC3 or further components also provide relevant information for the purpose of microclimate monitoring and to anticipate the formation of salt efflorescence. A statistically significant correlation was found between p[3] loadings corresponding to years 2008 and 2010, respectively (r = 0.75; p = 0.0003). However, the correlation is not significant if this comparison is conducted with p[4] or p[5] loadings (p > 0.05). This result suggests that PC3 accounts for systematic data variability that is maintained after two years of monitoring. In the 2008 and 2010 PCA models it was checked that sensors A and B are the ones with highest contribution in PC3. Both sensors are the ones located at a lower height from the floor, and consequently it is not surprising that their *RH* pattern appears as slightly different according to PCA. PC3 was disregarded because it does not explain *RH* data variability of sensors inserted into walls or frescoes. Thus, two components are enough for the purpose of the present study.

### 3.5. Recalibration of Sensors

Table 3 shows the average *RH* recordings of the four calibrated sensors and five sensors removed from the monitoring system, for each one of the 9 steps in the experiment. Taking into account that the mean value recorded from all calibrated sensors is 51.42, the table also shows the bias of each sensor. For the calibrated ones, this bias is about ±0.2%, while the bias of the other sensors is very similar except for G that is higher. Anyway, these values are much lower than the drift indicated by the manufacturer which is ±0.5% per year under normal storage conditions, which after the four years of monitoring would be ±2%. This bias is negative for sensors A, G and W, and positive for B and L. Taking into account that the five probes have been operative during four years, the low values of bias found in these sensors suggests that the drift has been negligible.

According to Figure 14, the difference of *RH* between sensors located in ribs with respect to wall sensors is about 6%, which is considerably higher than errors found in the recalibration experiment. Thus, the measurement accuracy seems adequate for the purpose of discriminating among sensors located in different positions of the vault and detecting those ones with an abnormal performance that could be caused by humidity problems.

Table 3 also indicates the average temperature recorded by sensors in this experiment. The mean temperature registered by calibrated sensors was 20.11 °C, resulting in a bias of about ±0.3. Similarly, the bias of sensors A, B, L, and W was +0.3, which is a similar value suggesting that the drift of temperature sensors has also been rather small. Sensor G seems to be slightly abnormal because its bias for temperature is 1.33 °C and also presents the highest bias for *RH*. The reason is unknown and further studies will be necessary to better investigate the aging and drift of all sensors.

## 4. Conclusions

A principal components analysis was carried out with *RH* data recorded in 2007, 2008 and 2010. It was seen that the performance of *RH* sensors installed in walls or frescos differs from those located on the ribs or at the cornice, which indicates that the monitoring system is accurate enough to assess water adsorption/desorption dynamics at the boundary layer. Moreover, a physical interpretation has been provided for PC1 and PC2.

Results confirm that PCA is a useful tool for the multivariate statistical analysis of data from *RH* sensors installed for the microclimate monitoring of fresco paintings. The loading plot corresponding to PC1 and PC2 characterizes the similarities and dissimilarities among all *RH* sensors.

A direct relationship has been observed between abnormal p[2] values in sensor AB and the formation of salt efflorescence in the area where this sensor is located, which reflects that this monitoring system is useful for the preventive conservation of fresco paintings. Data recorded in the next years will be analyzed using the same approach in order to diagnose the presence of sensors with abnormally high values or with a clearly different pattern, which might reflect humidity problems caused by rainwater infiltration.

In this paper we conclude that is possible to monitor frescoes with modern humidity sensors that do not need frequent calibrations as in previous decades. With this long-term data acquisition system we can use PCA for identifying abnormal conditions of the paintings. This PCA can detect an abnormal performance in one sensor that might correspond to a failure of the monitoring system [32] or a change in the microclimatic conditions surrounding that sensor. The last case was detected at the renaissance frescoes of the Valencian’s cathedral and the system described in this paper seems to be a good tool for preventing these situations.

This is the first time that PCA is used for preventive conservation with a satisfactory result and a practical interpretation was provided for its second component. We have demonstrated that the use of the humidity sensors and the interpretation of the first two components of the PCA can be a very powerful tool for preventive conservation of frescoes.

## Figures and Tables

**Table 1.** Sensors providing *RH* and T^a^ data appropriate for the multivariate statistical analysis.

		**YEAR**					
		**2007**		**2008**		**2010**	
**Position**	**Sensor^a^**	RH	T^a^	RH	T^a^	RH	T^a^
Cornice	A, B	OK^b^	OK	OK	OK	OK	OK
Ribs	C, D, I, J, X	OK	OK	OK	OK	OK	OK
	S	OK	OK	X	OK	OK	OK
	Ñ	X	OK	X	OK	X	X
Walls	L, P, V	OK	OK	OK	OK	OK	OK
	F	X	OK	X	OK	X	OK
	M	X	OK	X	OK	X	X
	AA	OK	OK	OK	X	OK	OK
	G, Z	OK	OK	OK	OK	X	X
	U	OK	OK	OK	OK	OK/X^c^	OK
	Q	OK	OK	X	OK	OK	OK
Paintings	E, K, O, R, T, W	OK	OK	OK	OK	OK	OK
	H, Y	OK	OK	OK	OK	X	X
	N	X	X	X	OK	X	X
	AB	OK	OK	OK	X	X^d^	OK

a Same code as in Figure 3.

b OK: operative sensors with a correct performance. X: non-operative sensors or with a very abnormal performance.

c Sensor U was operative in 2010 up to July 18th. Since then, it recorded abnormal data (negative values between −17 and −23).

d Sensor AB was discarded in 2010 because its performance was different from the sensors located on the paintings.

**Table 2.** Number of days in which the *RH* and T^a^ mean of sensors was out of the recommended range (285 days were monitored in 2008 and 295 in 2010)

				**Days out of range**
**Frescoes**	**Temperature**	**>25 °C**	2008	92
			2010	119
	**Relative humidity**	**>60%**	2008	117
			2010	86
		**<45%**	2008	21
			2010	35
**Air**	**Temperature**	**>25 °C**	2008	86
			2010	115
	**Relative humidity**	**>60%**	2008	56
			2010	47
		**<45%**	2008	54
			2010	81

**Table 3.** Results for *RH* recalibration conducted in August 2011 for 5 sensors installed in 2007 with respect to 4 sensors calibrated by the manufacturer. All sensors were introduced in a climatic chamber with *RH* increasing in 9 steps from about 15% up to 80%.

	**CALIBRATED SENSORS**				**SENSORS FOR RECALIBRATION**				
**STEP**	S1	S2	S3	S4	A	B	L	G	W
**1**	16.95	16.89	17.14	17.16	17.82	17.85	18.60	17.82	17.87
**2**	26.80	26.50	26.79	26.87	27.12	27.34	27.96	27.27	27.17
**3**	36.82	37.03	36.92	37.00	37.16	37.62	38.14	37.24	37.46
**4**	42.36	42.52	42.74	42.50	42.37	42.86	43.01	42.18	42.68
**5**	53.29	53.60	53.44	53.60	53.40	53.41	53.58	52.48	53.25
**6**	59.40	59.21	59.31	59.48	58.81	59.22	58.77	57.71	58.53
**7**	69.83	70.24	70.10	70.43	69.58	69.87	69.05	68.37	69.15
**8**	75.18	75.30	75.94	75.88	75.44	75.40	74.57	73.98	74.42
**9**	80.01	80.61	81.69	81.68	80.83	80.92	79.66	79.69	79.79
**MEAN HR**	51.18	51.32	51.56	51.62	51.39	51.61	51.48	50.75	51.15
**MEAN HR—51.42%**	−0.24	−0.10	0.14	0.20	−0.03	0.19	0.06	−0.67	−0.27
**T^a^ MEAN**	20.46	19.86	19.98	20.14	20.25	20.34	20.43	21.44	20.24
**T^a^ MEAN −20.11**	0.35	−0.25	−0.13	0.03	0.14	0.23	0.32	1.33	0.13
