# Correction: Zarzo, M. *et al*. Long-Term Monitoring of Fresco Paintings in the Cathedral of Valencia (Spain) through Humidity and Temperature Sensors in Various Locations for Preventive Conservation. *Sensors* 2011, *11*, 8685-8710

**DOI:** 10.3390/s130405403

**Published:** 2013-04-22

**Authors:** Manuel Zarzo, Angel Fernández-Navajas, Fernando-Juan García-Diego

**Affiliations:** 1 Department of Applied Statistics, Operations Research and Quality, Universidad Politécnica de Valencia, Camino de Vera s/n, 46022 Valencia, Spain; E-Mail: mazarcas@eio.upv.es; 2 Department of Applied Physics (UD Agriculture Engineering), Universidad Politécnica de Valencia, Camino de Vera s/n, 46022 Valencia, Spain; E-Mail: afnavajas@fis.upv.es

A typo has been found in our paper [1]. It is stated on page 8698 that the range of acceptable temperature for the conservation of frescoes is 6–25 °C while in the case of RH, this range is 45%–60%, according to the Italian Standard UNI 10829 (1999). These recommended ranges of temperature (6–25 °C) and RH (45%–60%) correspond to the Standard DM 10/2001 [2], not to UNI 10829. The authors would like to apologize for any inconvenience this may have caused to the readers of this journal.
